# CD123 Is Consistently Expressed on *NPM1*-Mutated AML Cells

**DOI:** 10.3390/cancers13030496

**Published:** 2021-01-28

**Authors:** Vincenzo Maria Perriello, Ilaria Gionfriddo, Roberta Rossi, Francesca Milano, Federica Mezzasoma, Andrea Marra, Orietta Spinelli, Alessandro Rambaldi, Ombretta Annibali, Giuseppe Avvisati, Francesco Di Raimondo, Stefano Ascani, Brunangelo Falini, Maria Paola Martelli, Lorenzo Brunetti

**Affiliations:** 1 Department of Medicine and Surgery, University of Perugia, 06131 Perugia, Italy; vincenzomaria.perriello@unipg.it (V.M.P.); ilaria.gionfriddo@unipg.it (I.G.); roberta.rossi@unipg.it (R.R.); francesca.milano@unipg.it (F.M.); federica.mezzasoma@unipg.it (F.M.); andrea.marra@studenti.unipg.it (A.M.); stefano.ascani@unipg.it (S.A.); brunangelo.falini@unipg.it (B.F.); 2 Azienda Socio-Sanitaria Territoriale Papa Giovanni XXIII, 24127 Bergamo, Italy; ospinelli@hpg23.it (O.S.); arambaldi@asst-pg23.it (A.R.); 3 Department of Oncology and Hematology, University of Milan, 20122 Milan, Italy; 4 Hematology and Stem Cell Transplant Unit, Campus Biomedico University Hospital, 00128 Rome, Italy; o.annibali@unicampus.it (O.A.); g.avvisati@unicampus.it (G.A.); 5 Hematology and Bone Marrow Transplant Unit, Catania University Hospital, 95125 Catania, Italy; diraimon@unict.it; 6 Hematology and Bone Marrow Transplant Unit, Santa Maria della Misericordia Hospital, 06131 Perugia, Italy; 7 Pathology, Santa Maria Hospital, 05100 Terni, Italy

**Keywords:** acute myeloid leukemia (AML), CD123, NPM1, FLT3, immunotherapy

## Abstract

**Simple Summary:**

One-third of adult acute myeloid leukemia (AML) harbors *NPM1* mutations. A deep knowledge of the distribution of selected antigens on the surface of *NPM1*-mutated AML cells may help optimizing new therapies for this frequent AML subtype. CD123 is known to be expressed on leukemic cells but also on healthy hematopoietic and endothelial cells, although at lower levels. Differences in antigen densities between AML and healthy cells may enlighten therapeutic windows, where targeting CD123 could be effective without triggering “on-target off-tumor” toxicities. Here, we perform a thorough analysis of CD123 expression demonstrating high expression of this antigen on both *NPM1*-mutated bulk leukemic cells and CD34^+^CD38^−^ cells.

**Abstract:**

*NPM1*-mutated (*NPM1*mut) acute myeloid leukemia (AML) comprises about 30% of newly diagnosed AML in adults. Despite notable advances in the treatment of this frequent AML subtype, about 50% of *NPM1*mut AML patients treated with conventional treatment die due to disease progression. CD123 has been identified as potential target for immunotherapy in AML, and several anti-CD123 therapeutic approaches have been developed for AML resistant to conventional therapies. As this antigen has been previously reported to be expressed by *NPM1*mut cells, we performed a deep flow cytometry analysis of CD123 expression in a large cohort of *NPM1*mut and wild-type samples, examining the whole blastic population, as well as CD34^+^CD38^−^ leukemic cells. We demonstrate that CD123 is highly expressed on *NPM1*mut cells, with particularly high expression levels showed by CD34^+^CD38^−^ leukemic cells. Additionally, CD123 expression was further enhanced by *FLT3* mutations, which frequently co-occur with *NPM1* mutations. Our results identify *NPM1*-mutated and particularly *NPM1/FLT3* double-mutated AML as disease subsets that may benefit from anti-CD123 targeted therapies.

## 1. Introduction

Acute myeloid leukemia (AML) is an aggressive cancer of hematopoietic stem and progenitor cells (HSPCs) [1], affecting almost 20,000 individuals every year in Europe [2]. Despite AML prognosis has significantly improved in recent years, 60% to 70% of patients diagnosed with AML eventually die of leukemia [1]. Such poor prognosis is mainly due to the high incidence of AML relapse after conventional treatment [3].

A significant fraction of AML relapses is thought to be secondary to the persistence of residual leukemic cells, able to re-establish the full tumor bulk [4]. In this regard, previous works suggest that leukemic cells with a stem-like phenotype (leukemic stem cells; LSCs) tend to survive chemotherapy, playing a major role in AML relapse [5,6]. Indeed, larger LSC pools at diagnosis are predictive of chemoresistance and worse prognosis [7].

Novel approaches aimed to eradicate residual disease in AML are under development [8,9]. Particularly, novel immunotherapeutic strategies targeting surface antigens are under investigation [10], with the hope to replicate the remarkable results of anti-CD19 bispecific antibodies and CAR-T cells in B-acute lymphoblastic leukemia (B-ALL) [11,12]. However, a major challenge for the clinical applicability of immunotherapy in AML is target selection, as no antigen that is selectively expressed on AML cells has been identified so far [13].

CD123, the alfa-subunit of the interleukin-3 (IL-3) receptor, is an attractive target, which has been reported to be expressed by the majority of AML patients, both on bulk leukemic cells and CD34^+^CD38^−^ putative LSCs [14,15]. However, CD123 expression on normal HSPCs and endothelial cells still represents a potential threat for “on-target off-tumor” effects, including myelosuppression and vascular toxicities, especially for powerful antigen-sensitive strategies such as Chimeric Antigen Receptor T (CAR-T) cells [16,17]. We believe that identifying AML subgroups with the highest CD123 expression on AML cells and putative LSCs may open therapeutic windows where anti-CD123 immunotherapy could be effective without causing major toxicities on CD123-positive vital tissues.

In the past years, several groups have investigated the landscape of CD123 expression in AML. However, the majority of studies have evaluated CD123 expression only on bulk and CD34^+^ cells [18,19], while CD34^+^CD38^−^ cells have been analyzed only in a relatively small number of patients [20,21,22]. Moreover, whether CD123 expression is higher on CD34^+^CD38^−^ than bulk AML cells and whether it correlates with specific risk categories is still controversial [15,20].

About one-third of adult AML patients harbor *NPM1* mutations [23]. Specific clinical and pathologic features granted *NPM1*-mutated AML the designation as a distinct entity of the World Health Organization classification of hematopoietic tumors [24]. Although large clinical studies have demonstrated that *NPM1*-mutated AML has a relatively favorable prognosis, about 50% of patients eventually die due to relapse and disease progression [25,26]. The prognosis is even poorer when *FLT3* internal tandem duplications (*FLT3*-ITD) coexist [27]. Interestingly, a previous work from our group suggested that *NPM1*mut putative LSC express CD123 [28]. Although several studies have described CD123 expression in large cohorts that included *NPM1*mut patients, no study had been designed to specifically analyze this AML subgroup or to compare CD123 fluorescence intensities between bulk AML cells and the CD34^+^CD38^−^ population [13,15,19].

Here, we investigate CD123 expression in a large number of newly diagnosed AML, focusing on the correlation between CD123 expression and *NPM1* mutational status, with the aim to explore whether *NPM1*mut AML could represent an entity that could particularly benefit from anti-CD123 therapies.

## 2. Results

### 2.1. Study Population and Analysis

Between October 2010 and October 2020, 151 samples (74 bone marrows and 77 peripheral blood) from 80 female and 71 male adult patients with newly diagnosed AML (median age at diagnosis 60, range 22–90) were studied.

CD123 expression was studied by multiparameter flow cytometry on bulk leukemic cells in all samples. Additionally, we also analyzed CD123 levels on CD34^+^CD38^−^ putative LCS in 115 samples containing at least 50 events in this rare subpopulation. CD123 expression levels were reported as the percent of positive cells (PPC) and as the median fluorescence intensity (MFI). PPC was available for all 151 samples, while MFI for 123 samples (see Methods). An arbitrary cut-off of 20% PPC was set to assign CD123 positivity. A summary of all results is presented in Table 1.

### 2.2. CD123 MFI Is Higher on Putative CD34^+^CD38^−^ AML LSCs

**Figure 1:**
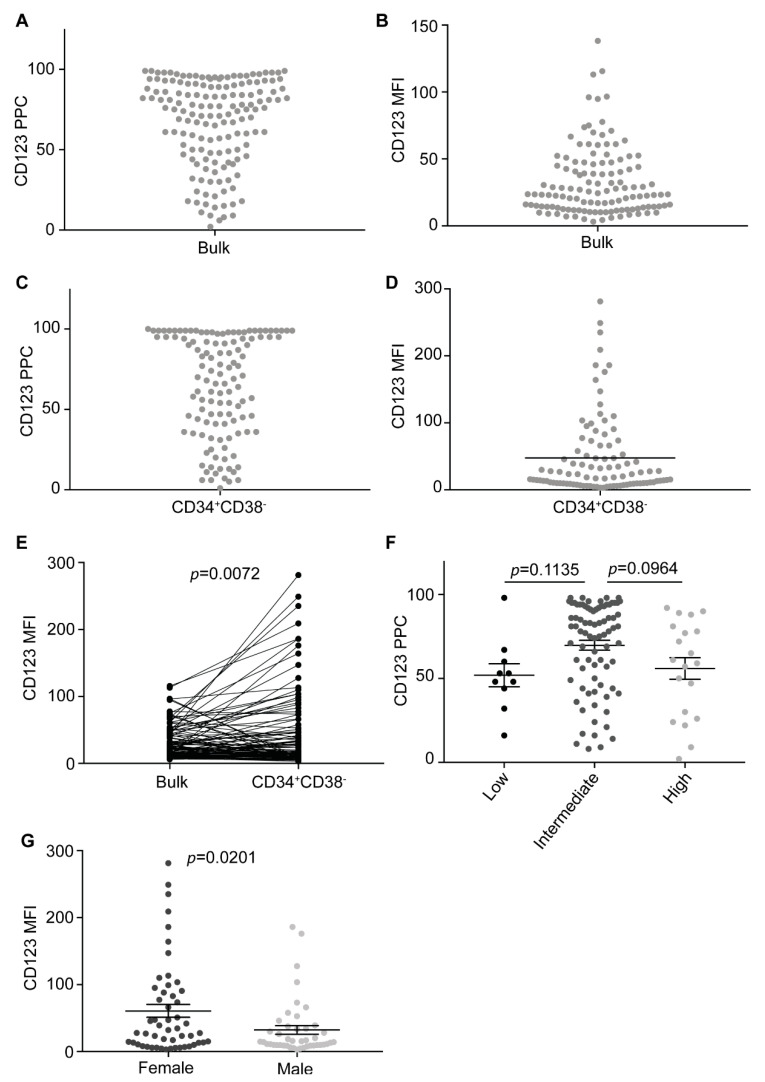
CD123 is ubiquitously expressed in acute myeloid leukemia (AML). (**A**) CD123 percent of positive cells (PPCs) on bulk cells in all samples (*n* = 151). (**B**) CD123 mean fluorescence intensities (MFIs) on bulk cells in all samples available (*n* = 122). (**C**) CD123 PPCs on CD34^+^CD38^−^ cells in all samples available (*n* = 119). (**D**) CD123 MFIs on CD34^+^CD38^−^ cells in all samples available (*n* = 96). (**E**) CD123 MFIs on bulk cells compared to CD34^+^CD38^−^ cells at diagnosis in all samples available (*n* = 96). Paired *t*-test. (**F**) CD123 PPCs on bulk cells sorted based on the cytogenetic risk (*n* = 111). Cytogenetics characteristics of all patients are reported in Table S1. Multiple comparison test. (**G**) CD123 MFIs on CD34^+^CD38^−^ cells in female and male patients (*n* = 96). Unpaired *t*-test. Bars represent mean and standard error.

We first analyzed CD123 expression on bulk cells in all samples. As previously reported by others [14,15,18,19], the vast majority of cases resulted CD123-positive (138/151, 91%). However, variable expression levels were observed among positive cases (mean PPCs 73 ± 22 and mean MFI 35 ± 26) (Figure 1A,B). We then looked into CD34^+^CD38^−^ cells, finding frequencies of CD123 positivity similar to those observed in bulk cells (mean PPCs 68 ± 30 and mean MFI 48 ± 60) (Figure 1C,D). Although no statistically significant difference was found between bulk and CD34^+^CD38^−^ CD123 PPCs, CD123 MFI was higher in CD34^+^CD38^−^ than in bulk cells (*p* = 0.0072) (Figure 1E), indicating that LSCs tend to have higher CD123 expression levels.

No statistically significant differences were found according to the cytogenetic risk stratification [27]. However, a clear trend towards higher CD123 expression levels was detected in the intermediate risk group (Figure 1F). Surprisingly, we also found a clear trend towards higher CD123 expression levels (MFI *p* = 0.00181 and PPC *p* = 0.0938) in female patients as compared to males when analyzing CD34^+^CD38^−^ LSC (Figure 1G).

### 2.3. CD123 Expression Is Consistently High in NPM1mut AML LSCs

**Figure 2:**
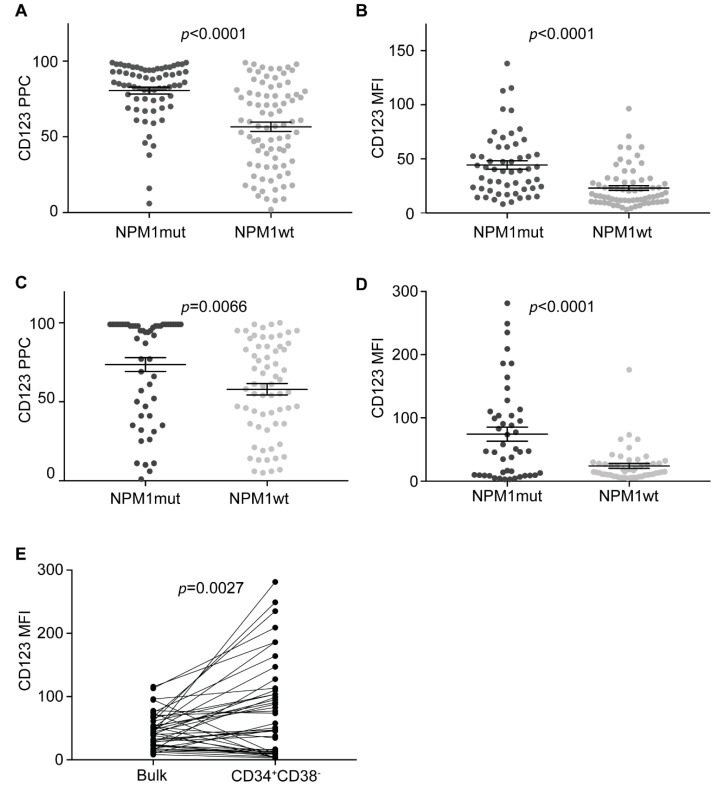
CD123 is highly expressed on *NPM1*mut AML cells. (**A**) CD123 PPCs on *NPM1*mut and *NPM1*wt AML bulk cells in all samples (*n* = 151). Unpaired *t*-test. (**B**) CD123 MFIs on *NPM1*mut and *NPM1*wt AML bulk cells in all samples available (*n* = 122). Unpaired *t*-test. (**C**) CD123 PPCs on *NPM1*mut and *NPM1*wt CD34^+^CD38^−^ cells in all samples available (*n* = 119). Unpaired *t*-test. (**D**) CD123 MFIs on *NPM1*mut and *NPM1*wt CD34^+^CD38^−^ cells in all samples available (*n* = 96). Unpaired *t*-test. (**E**) CD123 MFIs on *NPM1*mut bulk AML cells compared to CD34^+^CD38^−^ cells at diagnosis in all samples available (*n* = 44). Paired *t*-test. Bars represent mean and standard error.

As previous xenograft experiments suggest that *NPM1*mut AML LSCs are CD123-positive [28], we investigated CD123 expression in *NPM1*mut samples. *NPM1* was mutated in 68/151 patients (45%), and 97% (66/68) *NPM1*mut samples were CD123-positive. When compared with *NPM1*wt samples, *NPM1*mut cases displayed significantly higher CD123 expression levels on bulk AML cells (PPC and MFI *p* < 0.0001) (Figure 2A,B).

We then looked at CD123 expression in *NPM1*mut CD34^+^CD38^−^ AML putative LSCs. CD123 was positive in 91% of *NPM1*mut samples (49/54). Both PPCs and MFIs were significantly higher in *NPM1*mut putative LSC compared to *NPM1*wt samples (PPCs *p* = 0.0066 and MFI *p* < 0.0001) (Figure 2C,D). Average CD123 MFI was higher in CD34^+^CD38^−^ putative LSC than in bulk *NPM1*mut AML cells (*p* = 0.0029), confirming that CD123 is highly expressed on the surface of *NPM1*mut AML LSCs (Figure 2E).

**Figure 3:**
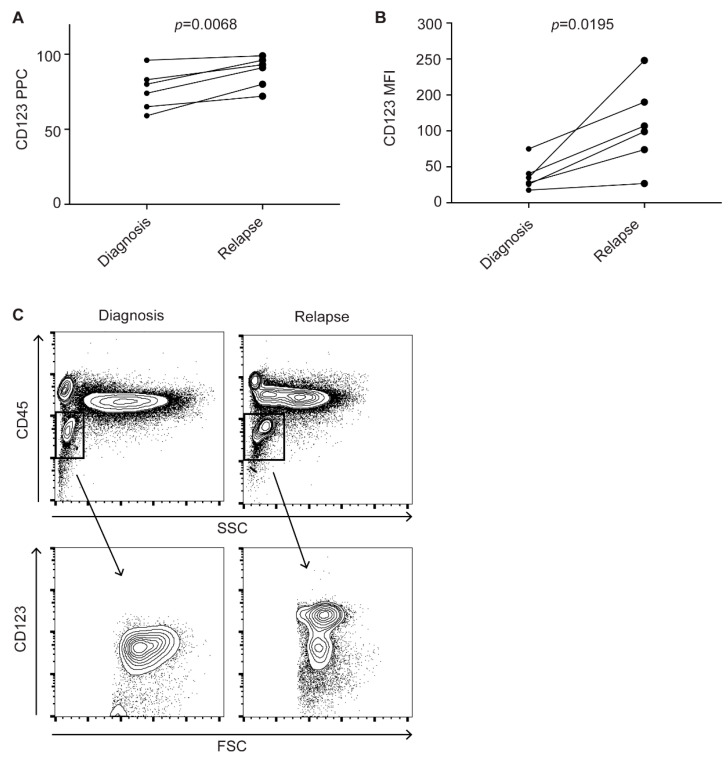
CD123 expression increases in relapsed *NPM1*mut AML. (**A**) CD123 PPCs on bulk *NPM1*mut AML cells at diagnosis and relapse (*n* = 6). Paired *t*-test. (**B**) CD123 MFIs on bulk *NPM1*mut AML cells at diagnosis and relapse (*n* = 6). Paired *t*-test. (**C**) Flow cytometry dot plots showing an increase of CD123 expression at relapse (right plots) compared to diagnosis (left plots) in a representative case of *NPM1*mut AML.

As several studies suggest that relapse frequently derives from residual LSCs after treatment [4,5], we analyzed CD123 expression on bulk AML cells in six *NPM1*mut patients at diagnosis and relapse. In all patients, CD123 expression increased at relapse, compared to diagnosis (PPC *p* = 0.0068 and MFI *p* = 0.0195) (Figure 3A–C). As two out of six patients had too-small numbers of CD34^+^CD38^−^ cells at diagnosis (<50 events), no comparison between diagnosis and relapse was possible in this subpopulation. Nonetheless, all six samples showed very high CD123 expression levels on CD34^+^CD38^−^ cell at relapse (average PPC 96 and average MFI 139). Altogether, these data confirm that CD123 is consistently expressed on *NPM1*mut AML putative LSCs.

### 2.4. CD123 Is Highly Expressed on FLT3-Mutated AML

**Figure 4:**
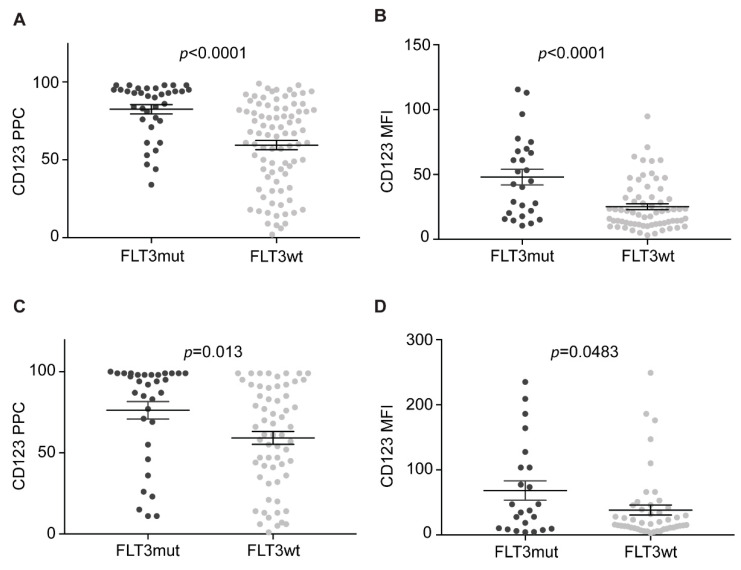
CD123 expression on *FLT3*mut and *FLT3*wt AML cells. (**A**) CD123 PPCs on *FLT3*mut and *FLT3*wt AML bulk cells in all samples available (*n* = 112). Unpaired *t*-test. (**B**) CD123 MFIs on *FLT3*mut and *FLT3*wt AML bulk cells in all samples available (*n* = 93). Unpaired *t*-test. (**C**) CD123 PPCs on *FLT3*mut and *FLT3*wt CD34^+^CD38^−^ cells in all samples available (*n* = 94). Unpaired *t*-test. (**D**) CD123 MFIs on *FLT3*mut and *FLT3*wt CD34^+^CD38^−^ cells in all samples available (*n* = 70). Unpaired *t*-test. Bars represent mean and standard error.

Since AML with mutated *NPM1* frequently harbors *FLT3* mutations, which have been previously reported to be associated with higher CD123 expression [29,30], we also analyzed the *FLT3* mutational status in 122 samples. *FLT3* was mutated in 46% (24/52) *NPM1*mut and in 19% (13/70) of *NPM1*wt cases. Two patients were positive for the *FLT3*-D835 mutation, while all others harbored *FLT3*-ITD. We first compared CD123 in *FLT3*-mutated (*FLT3*mut) and *FLT3* wild-type (*FLT3*wt) samples. *FLT3*mut samples showed significantly higher CD123 levels than *FLT3*wt cases. This was true for bulk cells (PPC *p* < 0.0001 and MFI *p* < 0.0001) and, though with a lesser degree of certainty, for CD34^+^CD38^−^ cells (PPC *p* = 0.013 and MFI *p* = 0.0483) (Figure 4A–D).

### 2.5. NPM1 and FLT3 Mutations Cooperate in Promoting CD123 Expression 

**Figure 5:**
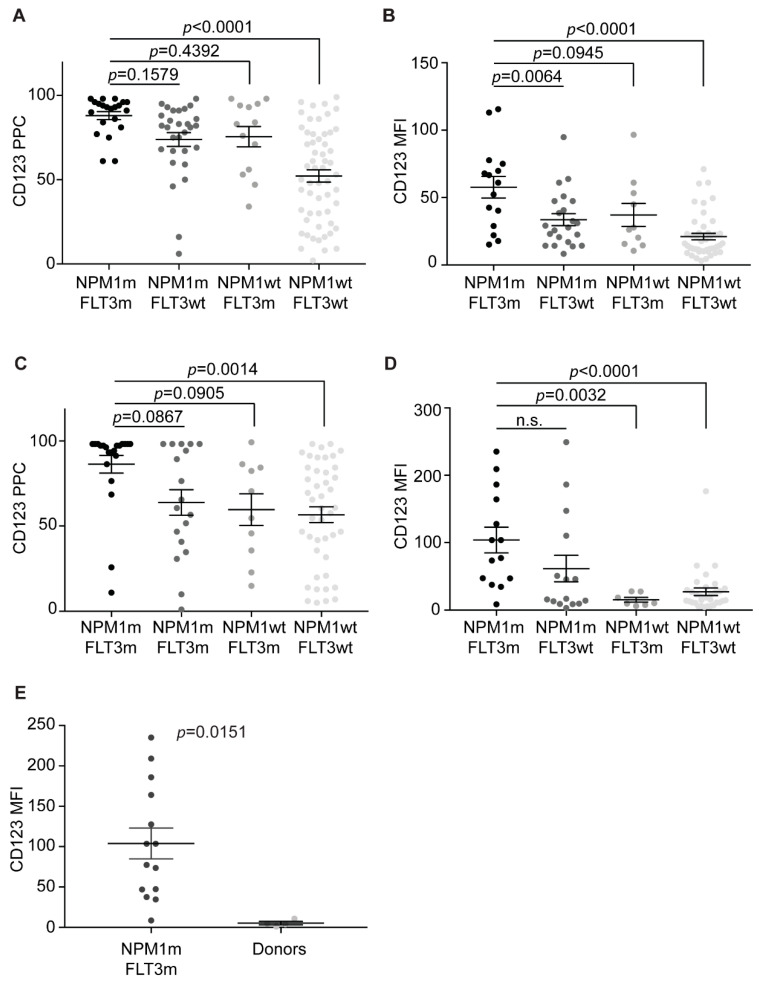
*NPM1*mut/*FLT3*-ITD AML LSC express the highest CD123 levels. (**A**) CD123 PPC on bulk AML cells, according to the *NPM1* and *FLT3* mutational status. Multiple comparisons test. (**B**) CD123 MFI on bulk AML cells, according to the *NPM1* and *FLT3* mutational status. Multiple comparison test. (**C**) CD123 PPC on CD34^+^CD38^−^ AML cells, according to the *NPM1* and *FLT3* mutational status. Multiple comparison test. (**D**) CD123 MFI on CD34^+^CD38^−^ AML cells, according to the *NPM1* and *FLT3* mutational status. Multiple comparison test. (**E**) CD123 MFI on *NPM1*mut*/FLT3*-ITD CD34^+^CD38^−^ AML cells and CD34^+^CD38^−^ cells from healthy donors. Unpaired *t*-test. NPM1m, *NPM1*mut. FLT3m, *FLT3*-ITD. Bars represent mean and standard error.

We then analyzed the CD123 expression in each of four the different *NPM1*/*FLT3*-ITD genotypes. The *NPM1*mut/*FLT3*-ITD, *NPM1*mut/*FLT3*wt, *NPM1*wt/*FLT3*-ITD, and *NPM1*wt/*FLT3*wt genotypes accounted, respectively, for 21/120 (18%), 29/120 (24%), 13/120 (11%), and 57/120 (47%). The highest CD123 expression was detected in double-mutated patients, while *NPM1*wt/*FLT3*wt cases displayed the lowest expression levels (Figure 5A,B), and *NPM1*mut/FLT3wt and *NPM1*wt/*FLT3*-ITD patients had intermediate CD123 (Figure 5A,B). Similar results were obtained studying either PPCs or MFIs in either bulk cells or in CD34^+^CD38^−^ subpopulations (Figure 5C,D). These results strongly suggest that *NPM1* mutations and *FLT3*-ITD cooperates in promoting CD123 expression.

### 2.6. CD123 Expression on HSPCs Is Significantly Lower than NPM1mut/FLT3-ITD CD34^+^CD38^−^ Cells

To compare the CD123 expression on LSC to that of normal progenitors, we analyzed the CD123 expression on bone marrow immature cells and on CD34^+^CD38^−^ cells from four healthy donors. Overall, healthy immature cells displayed very low CD123 levels (Table 1). Specifically, CD45^dim^-SSC^low^ cells showed a median value of CD123 PPCs of 12 and a median value of MFIs of 15, while CD34^+^CD38^−^ cells displayed even lower CD123 levels, with median values of CD123 PPC and MFI, respectively, of 3 and 4. With the caveat of the small sample size, we compared CD123 expression on CD34^+^CD38^−^ cells from healthy donors with CD34^+^CD38^−^ LSCs. As anticipated, the most significant difference was found with *NPM1*mut/*FLT3*-ITD CD34^+^CD38^−^ leukemic cells (MFI *p* = 0.0151) (Figure 5E), further confirming very high CD123 expression in this AML subset. 

## 3. Discussion

Immunotherapy targeting antigens expressed on AML cells represents a promising approach with the potential to reproduce the outstanding results achieved in B-ALL [31]. Despite several preclinical studies demonstrating the powerful antileukemic effects of bispecific antibodies and CAR-T cells targeting single AML antigens [32,33,34], only a few clinical trials are currently underway in AML patients [35]. Such a slow translation into clinical success is mainly due to the absence of targets with optimal expression profiles (i.e., highly expressed on neoplastic cells with low or no expression on vital healthy tissues) [10,13].

CD123 shows several features that prioritize this antigen among all promising potential target in AML. Previous studies have shown that the majority of AML patients express CD123 [13,15,18,19,20,22] and that higher CD123 expression seems to be associated with poorer prognosis [36,37,38]. Consequently, targeting CD123 represents an attractive approach to improve outcomes in AML.

However, CD123 expression on HSPCs and endothelium still represents a major obstacle [32]. Although CD123 expression on endothelial cells is very low [39], high levels of interferon-γ and tumor necrosis factor-α induced by cytokine release syndrome may facilitate CD123 expression and trigger capillary leak syndrome (CLS) [40]. This mechanism could explain why CLS occurred in two patients enrolled in a phase 1 first-in-human trial (NCT03203369 and NCT03190278) exploring the safety of a “universal” anti-CD123 CAR-T cell [41].

CD123 expression density is higher on AML cells than normal tissues. An important challenge is to find an optimal strategy where anti-CD123 CAR-T cells can still efficiently recognize leukemic cells while avoiding capillary leak syndrome and prolonged myelosuppression. Stevens et al. recently showed, by epitope density analysis, that anti-CD123 CAR-T cells could be able to eliminate tumor cells with higher CD123 antigen density, while sparing normal hematopoietic stem cells with lower antigen densities [42]. Arcangeli et al. confirmed the importance of CD123 antigen density and CAR affinity to fine-tune the level of anti-CD123 reactivity, merging efficacy with safety. In their study, the CAR-binding affinity was lowered in order to reduce the cytotoxicity against CD123^low^ endothelial cell lines while holding high activity against CD123^high^ AML cells lines [43]. In this context, identifying AML subsets with the highest CD123 expression levels seems relevant for the design of future clinical studies.

In this study, we demonstrate that CD123 is highly expressed on *NPM1*mut AML cells. CD123 expression on *NPM1*mut cells was already reported by our group while characterizing *NPM1*mut CD34^+^CD38^−^ leukemia-initiating cells in xenograft experiments [28]. Here, we show consistently high CD123 levels on putative *NPM1*mut CD34^+^CD38^−^ cells in patients, suggesting that anti-CD123 immunotherapies could be particularly effective in *NPM1*mut AML. The molecular mechanisms behind the higher CD123 expression and *NPM1* mutations in AML are unclear. However, NPM1 seems to play an important role in the differentiation of myeloid progenitors into mature myeloid cells through the IL-3/CD123/JAK2/STAT5 pathway [44,45,46]. Therefore, the interplay between *NPM1* mutation and high CD123 expression warrants further investigations.

CD123 expression was found to be particularly high in *NPM1*mut/*FLT3*-ITD samples. This was observed both on bulk cells, as well as in CD34^+^CD38^−^ cells. As *NPM1*mut/*FLT3*-ITD AML patients have relatively poor outcomes due to high relapse rates [26], we envision that anti-CD123 immunotherapy could be of particular benefit for this specific subset of patients.

We also found consistently higher CD123 levels at relapse when comparing CD123 expression in *NPM1*mut AML at diagnosis and relapse. We therefore hypothesize that CD123 plays a major role in the maintenance of this AML entity, given the importance of the IL-3 receptor for stem cell biology. This hypothesis is in line with several studies demonstrating the engraftment failure of CD123-negative AML-LSCs [20,47,48]. These results also suggest that CD123 may be a useful surface marker for measurable residual disease (MRD) monitoring by multiparameter flow cytometry. In this regard, the European LeukemiaNet recommends including CD123 while assessing MRD to identify early progenitors and LCSs [49]. Future studies will address the impact of CD123 in MRD monitoring of *NPM1*mut AML.

In conclusion, this work demonstrates that CD123 is highly expressed on the surface of *NPM1*mut AML cells both at diagnosis and relapse, with the highest levels detected in *NPM1*mut/*FLT3*-ITD samples. This study establishes *NPM1*mut AML as an attractive candidate for CD123-targeted therapeutic strategies laying the groundwork for the design of clinical trials in this specific AML subset.

## 4. Materials and Methods

### 4.1. Samples from AML Patients and Healthy Donors

BMs and PBs were collected in seven different Italian centers and subsequently analyzed in our laboratory within 24 h from sampling. In 6 cases, paired BM or PB samples were collected at both diagnosis and relapse. To compare CD123 expression in normal and pathologic samples, we also studied 4 bone marrows from healthy donors. All patients and donors gave their written informed consent before either BM aspiration or PB collection.

Routine morphologic, immunohistochemistry, cytogenetic, and molecular studies to assess *NPM1* and *FLT3* mutational status were performed as previously described [28]. *NPM1* mutations were also confirmed by Western blot analysis [50]. *NPM1* and *FLT3* mutational status was available, respectively, for 151/151 and 122/151 samples. In 111 cases, a successful karyotype analysis was obtained through standard G-banding [51]. Cytogenetic risk was defined according to the European LeukemiaNet 2017 recommendations, as previously reported [27]. Cytogenetics characteristics are reported in Supplementary Table S1.

### 4.2. Flow Cytometric Immunophenotyping

Heparinized peripheral blood or bone marrow samples were lysed for erythrocytes and stained with predefined optimal concentrations of 4 antibodies: CD34-FITC, CD123-PE, CD45-PerCP, and CD38-APC (Becton Dickinson, San Diego, CA, USA). Blasts were gated as CD45^dim^-side scatter (SSC)^low^ cells in samples with no- or early myeloid differentiation (see, also, Figure 3C) and as CD45^dim-pos^-SSC^low-int^ in cases with myelomonocytic or monocytic differentiation. CD123 expression analysis was analyzed on bulk leukemic cells and CD34^+^CD38^−^ precursors.

To set the cut-off point to distinguish between CD123 negative and positive cells, we used the “Fluorescence Minus One” technique, as described by Perfetto et al. [52]. A single case was arbitrarily judged CD123-positive when the percentage of positive bulk cells was higher than 20%. In healthy controls, immature cells were selected as CD45^dim^-SSC^low^ and CD123 analysis was performed on both whole immature cell population and CD34^pos^CD38^neg^ precursors. Data were reported as both CD123 percentage of positive cells (PPC) and median fluorescence intensity (MFI).

In all specimens, cell doublets and debris were excluded from analysis by forward scatter (FSC) vs. SSC dot-plot examination. Analysis was performed on either a FACSCalibur or FACSCanto II flow cytometers using the CellQuest Pro 6.0 or FACS Diva 7.0 analysis software (BD Biosciences, Franklin Lakes, NJ, USA). PPC was determined on all samples (analyzed on FACSCalibur or FACSCanto II). MFI was also studied in all samples; however, only those samples acquired on FACSCalibur were reported (i.e., the vast majority), as MFI values are not comparable between these two machines (four decades vs. five decades).

### 4.3. Statistical Analysis

Statistical analysis was carried out using GraphPad Prism 7 (GraphPad Software, San Diego, CA, USA). Differences between two groups were determined using paired or unpaired two-tailed *t*-test, depending on the experiment performed (see figure legends for each individual experiment). Differences among three or more groups were compared with an unpaired ANOVA test performing post-hoc multiple comparison tests. In all tests, *p*-values were considered statistically significant if <0.05.

## 5. Conclusions

**Figure 6:**
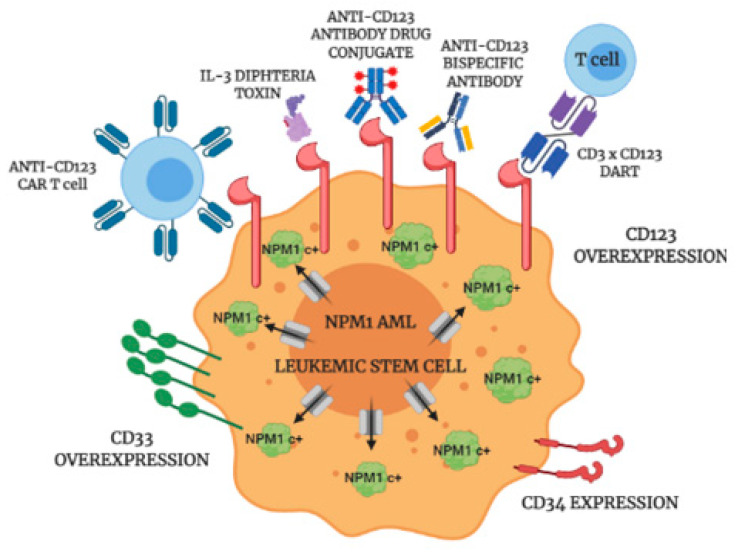
Anti-CD123 targeted therapies. Cartoon depicting the possible approaches to target CD123 on *NPM1*mut AML LSCs. NPM1c+, mutant NPM1; DART, dual affinity retargeting.

Our results suggest that *NPM1*mut AML could be particularly sensitive to anti-CD123 target therapies, for the differences in CD123 antigen densities between *NPM1*mut AML and healthy cells expressing low levels of CD123 (such as endothelial cells and HSCs) could reveal a therapeutic window where immunotherapeutic strategies could effectively eliminate *NPM1*mut LSCs (Figure 6) while avoiding capillary leak syndrome and prolonged pancytopenia. In conclusion, we believe that anti-CD123 immunotherapy holds promise for success in *NPM1*mut AML.

## Figures and Tables

**Table 1:** Summary of CD123 expression in all samples. Summary of CD123 expression studied by flow cytometry in bulk acute, myeloid leukiemia (AML) and CD34^+^CD38^−^ cells. * CD123 expression was studied in immature cells gated as CD45^dim^SSC^low^ cells (see Methods). LR, low risk; IR, intermediate risk; and HR, high risk. *p*-values are for unpaired *t*-tests, unless otherwise specified.

Subgroup	Bulk Cells CD123 PPC Median (25–75th Percentile), *n*	Bulk Cells CD123 MFI Median (25–75th Percentile), *n*	CD34^pos^CD38^neg^ CD123 PPC Median (25–75th Percentile), *n*	CD34^pos^CD38^neg^ CD123 MFI Median (25–75th Percentile), *n*
All samples	76 (48–91), 151	23.5 (13.7–46.3), 122	71 (41–95), 119	20 (9.7–66.1), 96
				
Female	78 (53.8–92), 80	29.1 (14.9–47.4), 68	84 (44.8–98), 60	30 (11.3–90), 52
Male	71 (46–86), 71	22.3 (13.4–38.6), 54	58 (35–91), 59	14.6 (9.6–36.6), 44
*p* value	0.2557	0.2883	0.0598	0.0201
				
LR cytogenetics	50.5 (41–61.8), 10	11.8 (11.8–25.5), 9		
IR cytogenetics	80 (49.5–93), 81	23.7 (14.3–51.6), 65		
HR cytogenetics	60 (27–80.3), 20	16 (10.5–35.3), 17		
*p* value (ANOVA)	0.0286	0.0455		
				
*NPM1*mut	84.5 (74.3–94), 68	39.8 (22.4–61), 54	94.5 (48.5–99), 54	47.4 (9.6–106.9), 45
*NPM1*wt	58 (32–79), 83	16.5 (10.7–28.4), 68	60 (36–85), 65	15 (9.9–29), 50
*p* value	<0.0001	<0.0001	0.0066	<0.0001
				
*FLT3*mut	91.5 (75.3–95), 36	43.8 (19.7–68.4), 26	93 (58.5–98.8), 32	37.5 (9.7–103.7), 23
*FLT3*wt	66.5 (38.3–82.3), 86	20.7 (11.8–32.5), 67	61.1 (35.8–90.3), 62	16.9 (11–41.8), 47
*p* value	<0.0001	<0.0001	0.013	0.0483
				
*NPM1*mut/*FLT3*-ITD	93 (82.5–96), 21	61 (28.9–75), 15	98 (89.5–99), 21	90.6 (44.7–169.5), 14
*NPM1*mut/*FLT3*wt	81 (67.8–89.5), 29	28.5 (16.3–47.4), 22	63.5 (39.5–99), 18	16.9 (9.7–110), 15
*NPM1*wt/*FLT3*-ITD	83 (54.5–94.5), 13	27 (15.4–55.2) 10	63 (32.8–85.5), 10	10.3 (7.6–27.6) 7
*NPM1*wt/*FLT3*wt	53 (28–77), 57	14.6 (10.3–23.5), 45	61.1 (33–84.5), 44	18.6 (8.6–29.4), 33
*p* value (ANOVA)	<0.0001	<0.0001	0.0029	<0.0001
				
Healthy donors	13 (5.25–16.25), 4 *	15.5 (6–20.8), 4 *	2.5 (1.3–8.3), 4	4.4 (1.9–9.5), 4

## Data Availability

Data available on request due to restrictions.

## Supplementary Materials

The following are available online at https://www.mdpi.com/2072-6694/13/3/496/s1, Table S1: Karyotyping results of all patients enrolled in the study.
